# Understanding the role of the art museum in teaching clinical-level medical students

**DOI:** 10.1080/10872981.2021.2010513

**Published:** 2021-12-05

**Authors:** Heather J. Kagan, Margot Kelly-Hedrick, Elizabeth Benskin, Suzy Wolffe, Melissa Suchanek, Margaret S. Chisolm

**Affiliations:** a Department of Medicine, Memorial Sloan Kettering Cancer Center, New York, NY, USA; b Duke University School of Medicine, Durham, NC, USA; c Department of Teaching and Learning, Baltimore Museum of Art, Baltimore, MD, USA; d Baltimore Museum of Art, Baltimore, MD, USA; e Department of Hematology, Albert Einstein College of Medicine, Bronx, NY, USA; f Department of Psychiatry and Behavioral Sciences, and Department of Medicine, Johns Hopkins University School of Medicine, Baltimore, MD, USA

**Keywords:** Arts and humanities, visual arts, art museum, professional identity formation, undergraduate medical education, clinical learners, medical students

## Abstract

Introduction. The role of the visual arts in medical education has been understudied, especially with regard to program evaluation and learner assessment of complex competencies such as professional identity, team building, and tolerance for ambiguity. We designed a study to explore how an integrative art museum-based program might benefit 3rd and 4th year medical students. Methods. We piloted 6 sessions with 18 participants. Evaluation methods included post-session surveys and semi-structured focus groups, which we qualitatively analyzed using an open-coding method. Results. Seven themes emerged from the analysis related to the overarching realms of ‘form’ and ‘function.’ ‘Form’ themes included structural elements of the sessions that enabled engagement: (1) group format, (2) methods (e.g., discussion prompts, activities), (3) setting (e.g., physical space of the museum, temporal space), and (4) objects (e.g., paintings, sculptures). ‘Function’ themes included the personal and professional value and meaning derived from the sessions: (1) appreciation of others, (2) critical skills, and (3) personal inquiry. Discussion. Our results expand what is known about the role of the visual arts in medical education by suggesting that the visual arts may facilitate clinically relevant learning across a range of competencies via specific formal aspects (group format, method, setting, objects) of art museum-based pedagogical methods.

## Introduction

Throughout the 20th century, amidst rapidly developing technological advances, medical schools and educators increasingly focused their teaching on the acquisition of scientific knowledge [1–3]. With this shift, medical learners moved further away from the bedside, become more specialized and isolated, and experienced unprecedented rates of burnout [4–6]. The integration of the arts and humanities – historically seen as fundamental to the practice of medicine – into medical education has been proposed as a solution to these problems. Reflective writing, creative writing, close looking of visual art, film viewing, music, history, dance, and theater arts have all been incorporated into medical education in a variety of ways, from brief independent sessions to longitudinal coursework[2]. Whether and how such integrative programs (i.e., programs that integrate the arts and humanities into medical education) benefit learners is not well known[7].

A recent scoping review identified thousands of publications on the role of integrative programs in medical education[8]. The review’s authors noted that this large literature lacks a coherent framework or narrative. In addition to this gap, the authors identified several others. They found relatively few programs that included clinical-level medical learners, incorporated the perspectives of non-medical personnel such as museum educators, or included program evaluation or learner assessments. As a potential way to address the need for a cohesive narrative for understanding the function of the arts and humanities in medical education, the scoping review authors proposed a conceptual framework called the Prism Model. This model is meant to guide educators in their design of integrative programs by asking them to consider an educational program’s objectives through four interrelated lenses (mastering skills, perspective taking, personal insight, and social advocacy) and to select teaching methods that best align with these objectives [9,10].

Among integrative arts and humanities programs, the role of the visual arts has been relatively understudied [2,8] The scant literature that does exist has focused primarily on medical students’ observational skills, with some evidence to suggest that visual arts-based methods can enhance attention to detail and improve physical exam skills [11–15]. Some studies have explored the role of close looking of visual art in enhancing medical trainee empathy and tolerance for ambiguity, key domains of clinical excellence, but more research is needed to further elucidate these findings [16–18]. Even less is known about the role of visual arts programs in a wider breadth of competencies and more complex aspects of learner development, including but not limited to professional identity formation, resilience, self-care, team building, critical thinking, communication, and social advocacy [2,11,19,20].

Now more than ever, in the ongoing wake of the coronavirus pandemic that has exacerbated health disparities with devastating effects, medical learners must become dynamic and creative clinicians who can meet unprecedented challenges [2,21,22]. While the arts and humanities hold promise for helping learners grow into clinicians who can face and address these difficult circumstances, more work needs to be done to understand exactly how the arts and humanities could achieve this goal. Our study explores how integrative visual arts programs, developed and led by an interdisciplinary team at a local art museum, may potentially benefit clinical-level medical students. We specifically explore students’ perception of educational structure and their perceived learning outcomes.

## Methods

### Participants

Eighteen 3rd and 4th year medical students at Johns Hopkins University participated in at least 1 of 6 unique sessions held at The Baltimore Museum of Art. We recruited participants by email, social media, flyers distributed on the medical school campus, and word of mouth. We provided participants with lunch and compensation for transportation.

### Art museum sessions

As part of the planning for a 4-week integrative arts and humanities course for medical students on human flourishing, we developed and evaluated 6 2-hour art museum-based sessions. We intended to pilot 8 sessions, but due to the coronavirus pandemic, we were unable to offer the final 2 sessions. Each session was piloted approximately monthly between September 2019 and March 2020. Two museum educators (EB, SW) and 1 medical educator (MC) co-designed and co-led all sessions.

**Figure 1.**
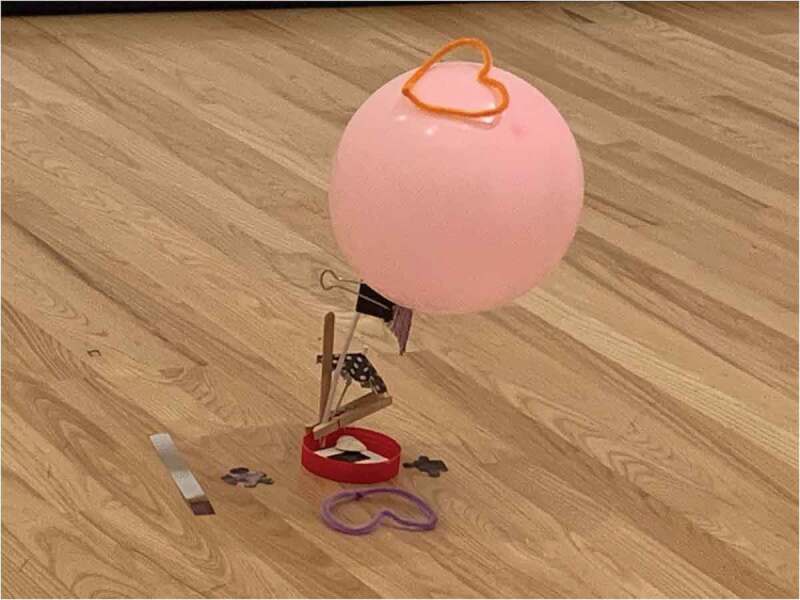
Example of a gift created by a participant for a relationship depicted in a painting viewed during Family session 2

Each session was unique and included 2–3 distinct activities designed around themes related to human flourishing (e.g., family, community, work/education) to align with the curricular objectives of the planned 4-week course (the course objectives as well as activities for each session are available as Appendix A) [23]. For example, during a family-theme session activity, participants selected a work that related to familial relationships and created a gift that this relationship might need (see Figure 1 for an example of what one participant created for this activity).

### Evaluation survey

Immediately following each of the 6 sessions, participants completed an anonymous paper survey. This post-session survey included 1 closed- and 3 open-ended items (the post-session survey is available as Appendix B). Seventeen of 18 (94%) participants responded to the closed-ended survey item, and 18 of 18 (100%) responded to the open-ended items.

### Focus groups

We conducted each of the 6 focus groups immediately following the completion of the post-session survey. The medical educator (MC) led the semi-structured focus group (the interview guide is available as Appendix C).

Each focus group lasted approximately thirty minutes. Responses were audio-recorded with verbal permission from the participants and sent to a secure transcription service, which provided a de-identified transcript of each session’s focus group (6 transcripts total). The study coordinator (MKH) entered all survey item responses and focus group transcripts into a secure, electronic database in preparation for analysis.

### Qualitative analysis

As our aim was to explore students’ perception of the educational structure of visual arts programming and their perceived learning outcomes, we utilized qualitative analysis to provide a thick description of this phenomenon. We performed a simple thematic qualitative analysis of both the open-ended survey item responses and focus group transcripts using a phasic method of coding and analysis[24]. Two reviewers (HK and MKH) independently generated themes from the open-ended survey item responses and focus group transcripts using open coding in Microsoft Word. We selected HK, who was not part of the session development or teaching team, and who was not present at any of the art museum sessions or focus group sessions, in an attempt not to further increase bias. After open coding on a subset of transcripts, the 2 independent codebooks were compared and consolidated into 1 codebook with the input of a 3rd reviewer (MC). The coders then returned to the data and coded the data independently. The coders then compared the codes and settled discrepancies with the 3rd reviewer. Final coding was completed in NVivo[25].

### Descriptive analysis

We performed a descriptive analysis of the 1 closed-ended survey item, which asked participants to rate the session’s overall educational value on a 4-point Likert scale: (1) ‘not valuable,’ (2) ‘somewhat valuable,’ (3) ‘valuable,’ and, (4) ‘very valuable.’

## Results

Descriptive analysis of the closed-ended survey item found that 16 of 17 (94%) respondents described their session as ‘3- valuable’ or ‘4- very valuable’ on a 4-point Likert scale.

In the thematic analysis of the focus groups and open-ended survey items, 2 overarching categories emerged: ‘form’ and ‘function.’ ‘Form’ themes relate to the structural elements of the session that the participants perceived as enabling their engagement, while ‘function’ themes relate to the value and meaning that the participants attributed to the sessions. Seven total themes became apparent within these overarching categories of form and function (Table 1). ‘Form’ themes include (1) group format, (2) method, (3) setting, and (4) objects; ‘function’ themes include (5) appreciation of others, (6) critical skills, and (7) personal inquiry.

1. **Group format**. This ‘form’ theme includes comments from participants on how the group experience was ‘really special’ compared to looking at art alone and allowed them to navigate ideas together in both pairs and larger groups. This theme also includes thoughts about group make-up, such as how different levels of trainees or participants from different backgrounds was ‘particularly useful.’
2. **Method**. This ‘form’ theme includes participants’ thoughts about the variety of learning experiences in the course. Sketching activities, open-ended questions, and discussions centering on artworks allowed participants to ‘explore [their] feelings’ in a ‘structured way.’
3. **Setting**. This ‘form’ theme includes participants’ descriptions of the physical museum space as ‘sacred.’ Participants felt that having ‘dedicated time’ away from their usual learning environment to think about topics like their fears in medicine was ‘really important.’ Participants noted that ‘because med school is very busy,’ this time and space for reflection was ‘something that we don’t necessarily take the time to do a lot.’
4. **Objects**. This ‘form’ theme captures participants’ reflections on art specifically as a way to broach sensitive topics, as ‘you can feel comfortable enough [to] share and bring what you need to bring without feeling like you, yourself, are under a microscope.’ Another participant commented on ‘an analogy between [arts-based activities] and patients,’ as art is communicative, adding that ‘it spoke back’ and ‘elicits this emotion from you.’
5. **Appreciation of others**. This ‘function’ theme includes participants’ thoughts on the value of diverse perspectives, community building, and understanding others. One participant reflected on how their group created a ‘variety of different narratives’ surrounding artworks and identified the limits of their own conditioned individual perspectives, which ‘helped [them] understand [them]selves and other people better.’ Another participant described how the activities created a powerful team with a unique ‘closeness’ they could rely on to mitigate one another’s’ fears about the medical profession, giving them the ‘opportunity to validate each other’ and ‘hear that other people shared similar fears.’ In considering an activity that asked participants to describe their partner’s interpretation of an art object to the larger group, they commented that ‘putting yourself in those shoes’ is directly applicable to medicine and useful for ‘helping patients [with] medical decision-making.’
6. **Critical skills**. This ‘function’ theme includes participants’ comments on critical thinking and dealing with discomfort and uncertainty. Participants noted experiencing ‘contradictory emotions or thoughts’ throughout the activities, prompting them to independently figure out ‘ways of just sitting with that dichotomy or somehow moving forward.’ One participant shared how they became more ‘comfortable with ambiguity,’ an experience which another credited as ‘the most beneficial in terms of growth for myself.’ Participants noted the relevance of ambiguity tolerance to medicine: ‘certainly pertinent for medicine because from a scientific perspective we don’t always have all the answers,’ and that practicing tolerating ambiguity could ‘sort of help quell some of the fears or the immediate kind of emotions that I might feel in the setting of a patient’s room.’ Participants reflected on how their own thought processes had become ‘formulaic’ to survive medical school, but valued the exposure to the different ways that artists and museum educators think. One remarked that ‘incorporating those into your own practice will let you answer some problems that you weren’t able to.’ Participants felt the arts-based activities honed their communication skills in a way that would prepare them to bridge knowledge gaps and ‘describe medicine or biology to a patient.’
7. **Personal inquiry**. This ‘function’ theme includes participants’ thoughts about big questions, including the meaning of life, art, what it means to be human, and what it means to be a physician. They pointed out that ‘the arts and the humanities are part of what give life meaning,’ and that while ‘there is no correct answer in art … that is very different from anything that I’ve understood in medical school.’ Participants focused on this in the context of medical practice, as physicians must also ‘keep in mind the meaning of it all when you’re seeing patients.’ They explained that they were able to ‘be more whole individuals’ and drill down to the question of, ‘why is what we do valuable?’

**Table 1.** Descriptions and exemplary quotes for the 7 themes that emerged from qualitative analysis

Theme Type	Theme	Description of theme	Exemplary quotes of theme from source data
Form	(1) Group format	Makeup and size of the group	‘The experience of working in my intimate group … was incredibly valuable’
	(2) Method	Teaching modality and method, variety of activities and tasks	‘The thing for me that … was really striking … is the way in which the questions were framed … it was just so open that it allowed us all to answer in our own ways and to be able to kind of have our own style in how we responded to things’
	(3) Setting	Physical museum space and temporal space (e.g., time for close looking)	‘Being in a museum, I felt like it tapped into my emotions’ ‘The architecture and the environment of the museum framed this experience’
	(4) Objects	Artworks as evocative objects, aesthetic features	‘The nice thing is that we were all looking at an object. The thing we were interpreting wasn’t located within us’
Function	(5)Appreciation of others	Diversity of perspective, cultivating empathy, team building	‘I was really pleasantly surprised by how … generative it was to hear other people’s perspectives on the objects we were looking at together that were very, obviously complementary, obviously rigorous and thoughtful, obviously beyond what I would’ve reached by myself’ ‘[It’s] very important to have those discussions and see you’re not the only person feeling this and being able to … validate each other’ ‘With a group of people you can add a lot of meaning to it and make it something really special’
	(6) Critical skills	Communication, critical thinking, dealing with unfamiliarity and uncertainty	‘ ….there’s a lot of information you can glean from nonverbal cues’ ‘You don’t have to have the answers but you can make hypotheses about what could be going on and you build evidence for those alternative hypotheses. And it’s just a process of critical thinking’
	(7) Personal inquiry	Making sense of meaning in life and professional identity, self-awareness and personal growth, empowerment, transformation, therapeutic effects	‘ … I think that taps into something deeper than just the cognitive thinking about what would be a good career. It makes you think deeper about what really speaks to my core. And that’s why I found the exercise really validating because what spoke to me in the art was also what I’m doing in my career’ ‘I think it helps maintain an intentional practice that’s consistent with my own values’

Participants emphasized the opportunities for self-awareness and personal growth in the activities and described these as ‘like a mirror was being held up.’ They expressed that the sessions challenged them to ‘critically think about [their] communication styles,’ and ‘maintain an intentional practice that’s consistent with [their] own values.’ Participants said they used their ‘creative thinking muscles’ and that the activities reinvigorated their imaginations. Participants also modeled vulnerability and empowered one other to share: ‘when you see other people step forward for something even if it’s not the same thing, sometimes you feel more empowered to step forward yourself,’ one said. One participant described the curative, therapeutic, and transformative effects of the experience as ‘awe-inspiring,’ ‘powerful,’ and emotionally evocative. Another mentioned gaining tools for self-care and learning how ‘to ensure that you’re able to maintain yourself throughout the process [of medical education].’

While each of these 7 themes has distinct features, participants frequently expressed complex ideas that cut across multiple themes. Comments frequently highlighted both form and function themes. Highlighting the themes of group format, appreciation of others, and critical skills, one participant observed:

It’s helpful that we have people from different backgrounds and community experiences. It was helpful to get those different perspectives. And, yeah, we shared our interpretations of the artworks and I think it helped me better appreciate the possible breadth of ways of interpreting or really anything, any situation.

Another participant commented:

You don’t have to have the answers but you can make hypotheses about what could be going on and you build evidence for those alternative hypotheses. And it’s just a process of critical thinking. There’s an element of imagination. But I think overall the theme of uncertainty, embracing it and feeling empowered by other people was what was most impactful for me today.

This highlights the two ‘function’ themes of critical skills and personal inquiry.

## Discussion

We offered 6 art museum-based sessions for 3^rd^ and 4^th^ year medical students and explored participants’ perceptions of how such sessions could be impactful to their medical education. Participants perceived the sessions as both personally and professionally valuable – helping them better understand themselves and navigate the world around them – as captured through the ‘function’ themes (Table 1). Participants thought the integration of the arts was fundamental to achieving these benefits, as reflected in the ‘form’ themes (Table 1). When describing the impact of the sessions, participants’ comments frequently highlighted themes of both form and function, revealing the inextricably interrelated nature of these themes, as visual art provides for unique functions and impact through its unique form. This echoes the concepts presented in the Prism Model, as the lenses offer different, but inherently interconnected, frameworks from which to teach[9].

As articulated by the Fundamental Role of the Arts and Humanities in Medical Education report issued by the Association of American Medical Colleges’ (AAMC), the arts and humanities have conceptual relevance to medical education in many ways, such as fostering tolerance to ambiguity, deepening learners’ understanding of patients’ stories, strengthening learners’ connections with patients to better serve them, and enhancing the well-being of budding physicians to provide them with tools to care for themselves and others. While research is lacking with regards to exactly how the arts and humanities can accomplish these aims and satisfy core medical school competencies, existing evidence demonstrates that the arts and humanities may help learners master skills, 1 of the 4 core lenses of the Prism Model: mastering skills, perspective taking, personal insight, and social advocacy [9,10,20,26]. The results of our exploratory qualitative study build on this and expand what we know about the role of the arts and humanities in medical education by suggesting that a visual arts program not only may enhance learning skills like observation, but also may benefit learners in deeper and more nuanced ways: appreciation of others and personal inquiry. These findings align with 2 other core lenses of the Prism Model: perspective taking and personal insight [9,10].

Our results suggest that participants perceive visual arts-based methods as useful for teaching many core competencies in medicine in a novel way compared to traditional scientific-based medical pedagogy. Participants saw the activities as relevant to providing compassionate and patient-centered care as captured by the ‘appreciation of others’ theme. They also perceived an impact in the domains of personal growth, honing self-care techniques, and increased tolerance of ambiguity and uncertainty. This supports both curative and intrinsic functions of the visual arts as described in the literature, which are directly applicable to practice-based learning competencies established by the Accreditation Council for Graduate Medical Education (ACGME)[20]. Participants appreciated a deepened awareness and progression of their professional identity, a critical aspect of medical education[19], as illustrated by the ‘personal inquiry’ theme. Finally, participants perceived higher-level development beyond observation skills, namely skills of tolerating ambiguity and communicating more effectively, as reflected in the ‘critical skills’ theme[16].

The novel ways in which participants felt that visual arts-based methods taught such competencies are reflected primarily through the ‘form’ themes, which echo previous observations that art’s inherent qualities lay the groundwork for engagement[27].. The ‘function’ themes reflected participants’ thoughts on how art supported meaning-making and higher-level skill development, roles for the visual arts which have been noted by others[27]. Our participants described deriving profound personal insight and novel perspective taking, core lenses of the Prism Model that in our work align with the extrapolated ‘personal inquiry’ and ‘appreciation of others’ themes, respectively. In extrapolating themes, it was often challenging to characterize a participant’s comment as a single theme, leading us to code many complex comments across multiple themes. This observation aligns with the emerging Prism Model that poses the arts and humanities as dynamic pedagogies requiring the analogy of a prism lens, as their benefits to learners are intertwined and overlapping.

Limitations of our study include confounding bias, inherently a risk with the design of our study without randomization as participants were recruited voluntarily and thus may be self-selected to inherently perceive benefit from an integrative visual arts program. Furthermore, while we can make conjectures about what outcomes participants might derive from such programming, we measured participants’ perceptions, an inherently subjective measure. Bias was also introduced by 2 of the qualitative reviewers’ presence in the museum sessions and focus groups. After data collection, in an attempt not to increase bias further, we added a third qualitative reviewer to the study team who was not a part of the sessions’ design, implementation, or data collection (HK). Another limitation is potential lack of replicability, given the high degree of museum and medical educator expertise (i.e., if replicated with another facilitator, results may be different) as well as the small number of qualitative analyzers and variability in content between art museum sessions.

Overall, our findings support the role of integrative arts and humanities programs in the education of 3rd and 4th year medical students, and suggest that the visual arts may facilitate clinically relevant learning of ACGME core competencies in ways that align with the AAMC Prism Model via specific formal aspects of art museum-based pedagogical methods (group format, method, setting, objects).

## Appendix A.

Course learning objectives and course activities

*Course objectives*

1. Facilitate deepened student reflection on what it means to be human, to be a physician, and to lead a good life (for oneself and one’s patients).
2. Facilitate student reflection on one’s sense of self in relation to one’s family, community, work, and education experiences.
3. Facilitate student reflection on how family, community, education, and work experiences offer opportunities for improving one’s life satisfaction and happiness, physical and mental health, character and virtue, meaning and purpose, and close social relationships.
4. Facilitate student reflection on the role of the arts and humanities in mastering skills, appreciating multiple perspectives, gaining personal insight, and supporting social advocacy.
5. Facilitate student reflection on how the arts and humanities can support self-care and wellbeing.

*Course activities*

**Family session 1**

- **Pair and share partner introductions**: Working in dyads, each person chooses an artwork that says something meaningful about themselves and discusses it with their partner. In the larger group, each person introduces their partner using the information they just learned. This activity takes place in a wing of the museum so pairs can wander the space.
- **Close looking**: Viewing Anthony Van Dyck’s painting, *Rinaldo and Armida*
  - Participants write down 10 things they see in the painting and share their list with a partner. Dyads consider what their partner helped them see that they had not on their own. Now dyads create a list of 10 things that neither partner had seen before.
  - Group discussion about how looking together was different than looking alone, followed by discussion on finding meaning in the artwork after the close looking exercise
- **Physical examination through sketching**: Viewing Wangechi Mutu’s sculpture *Water Woman*
  - Participants sit at different points around the sculpture and sketch it from their vantage point. Sketches are then shared.
  - The group reflects on the variety of valid perspectives about one thing and how this relates to medicine.
- **Artwork that speaks to the domain of family**: In the Contemporary Wing with many two-dimensional works covering the walls
  - Participants choose works in the gallery that speak to them about the domain of family. Artworks may be figural or abstract.
  - Participants reflect through writing, thinking about the ways in which the artwork speaks to them about family.
  - Participants are invited to share their reflections.

**Family session 2**

- **Circle of similarities and differences**:
  - Participants stand in a circle. The facilitator makes a series of statements and participants who identify with each statement step forward (e.g., ‘I am an only child,’ ‘I consider myself religious’). Afterwards participants reflect on what they noticed during the exercise.
- **Relationship activity**: This took place in the Contemporary Wing with multiple pieces of art
  - This exercise focuses on artworks that feature two figures in a dyadic relationship. Participants will write about the interrelationship of the subjects based on non-verbal cues.
- **Gift activity**: In the same room as previous exercise
  - Participants will each choose an artwork in the gallery that speaks to familial relationships. Using baggies of art materials, participants will create a gift (abstract or literal) that they think this relationship might need.
- **Disparate artworks**: Looking at two works—one by Nick Cave and one by Nari Ward
  - Students are asked to reflect on and discuss these questions: What are commonalities between these two disparate artworks? What does this exercise say about how people connect across difference? How might the activity inform participants’ thinking about a broader definition of family beyond the common understanding of the traditional nuclear family?

**Community session 1**:

- **Quilt activity**: Elizabeth Talford Scott quilt
  - Students spend time looking closely at the quilt. They write a sentence of what speaks to them about the quilt. Each student reads their sentence aloud, and then they work together to combine their sentences into a poem. Afterwards, they reflect on the process and its relevance to medicine.
- **Shinique Smith**: Community coming together through materials
  - Students observe, describe, and discuss the sculpture by Shinique Smith which includes pieces of clothing from friends, family, and acquaintances from across time and geographical location.
- **Sketching about community**
  - Students sketch and share one thing that they identify with more in one of their chosen communities. They then sketch an object that is related to their community of choice. Afterwards, students share their sketches and describe their chosen communities.
- **Reflection on 9/11 piece**: Viewing Jack White 9.11.01
  - Students comment on a large, multi-media piece about 9/11. They discuss the role of art in response to a community event.
- **Creating a community with objects**
  - Students are given a bag of scraps and materials (e.g., close pins, scraps of magazines, balloon, straw, strings of beads). They are asked to create a community from the objects.
  - Each student shares their community and discusses which objects were left out and why. Afterwards they group reflects on what defines a community.

**Community session 2**

- **Sketching about community**
  - Students are invited to think about a community of which they are a part. They draw 3–5 members of it symbolically. They volunteer to share their representations of community.
- **Sketching of figure**: Viewing D’mba, Baga
  - Students sketch the mask and reflect on how the sketching affects their perception of the object.
  - The museum educators provide some additional information on the figure—considered an ideal woman in the Baga community. Discuss how public masquerades were used to promote community expectations.
- **Return to first sketch**
  - Student look back on their first sketch and write in response to the following questions: In your community group what are the expectations of your behavior and who or what entity enforces them?

**Work and education session 1**

- **Quadrant activity about motivations for going into medicine**
  - Each student is given a sheet of paper which has four boxes, each with a prompt. In one gallery of the museum, they find a piece of art that relates to their motivation for entering the field of medicine, filling in each quadrant:
    - Quad 1: Sketch of piece
    - Quad 2: How does the piece relate to your motivations for medicine?
    - Quad 3: What is a song/piece of music that relates to this piece of art?
    - Quad 4: (Wait to fill out when together as group): note down similarities and differences, notes as others share
  - After students share their thoughts in front of their respective pieces, the exercise is repeated this time in a courtyard with a mosaic from the city of Antioch (in present-day Turkey). The same prompts are used, except this time students are asked to think about their *aspirations* for a career in medicine, filling out the same quadrants. Afterwards, students share and reflect.
- **Back to back drawing**
  - Students sit on stools back-to-back in front of a large painting. One student is facing away from the painting and has not seen it. The student facing the painting describes the painting to the student facing away, who has pencil and paper. The student facing away is not allowed to speak. The student facing the painting can’t see the drawing until the end.
  - The exercise is repeated with a new painting and the roles reversed.
  - The group discusses how the exercise relates to medicine.

**Work and education session 2**

- **Fears exercise**
  - Students start writing in response to the question: what is your greatest fear about challenges or failures in your future education or professional life? They are informed they won’t have to share their response.
  - Students spend 10 minutes silently exploring the outside of the large multi-media artwork made of fabric and paint by Katharina Grosse (*Is It You?*). As they walk through, they are asked: What do you see? What does it look like to you? What does it NOT look like? Does it remind you of anything?
  - The students are then invited to enter the interior of the fabric installation and reflect on the same questions for 10 minutes.
  - Afterwards, the group discusses the experience of exploring the interior and exterior of the installation and how it relates to their fears they wrote down at the start of the session.
- **Exploring doubts through video**
  - Start by viewing Howardena Pindell’s video *Free, White and 21*
  - Afterwards, the group discusses the artwork generally and then focuses on the figures that were seen in the video.
  - Then, they are invited to reflect on their lived experience and reflect on these questions: Can you think of a time when someone has blocked your path or doubted you? What inner voices did you hear? How did you persevere through the challenge? How important is it to feel as though you are seen and heard? If there comes a time that you as a practitioner cause others to feel ‘less than,’ what would you do?
- **Reflection on challenges and failures**: Transition to *Jo Smail: Flying with Remnant Wings*

1. In a gallery with multiple pieces of art, students are asked to reflect on their greatest fear about future challenges or failures and find a work of art that in some way makes you think differently about your greatest fear. Afterwards, the group convenes for those who want to share.

## Appendix B. Survey instrument

1. How valuable were each of the following activities in today’s session to you?

0--------------------------1--------------------------2--------------------------3

Not valuableSomewhat valuable Valuable Very valuable

1. Please describe an experience today that was especially meaningful for you (if you have specific feedback about any of the activities you may provide this here as well).
2. Do you have any comments about the logistics of today’s session?
3. What recommendations do you have for the 4-week elective that you haven’t already covered?

## Appendix C. Interview guide

For the purpose of this study, we will be creating and using audio recordings of the Focus Group. If you do not wish to be audio recorded, you may opt out of the Focus Group portion of this study.

1. Please describe for me what happened for you in today’s session.
2. What did you experience that makes you say that?
3. Please tell me more.
4. How is this relevant to medical education and/or practice?

How could even greater connections be made?
